# EJPT abstract book for Posttraumatic stress: state-of-the-art research and clinical implications for China

**DOI:** 10.3402/ejpt.v5.26477

**Published:** 2014-12-09

**Authors:** Miranda Olff, Zhonglin Tan

In a unique collaboration between The Seventh Hospital of Hangzhou, the International Society for Traumatic Stress Studies (ISTSS), and the Zhejiang Behavior Medicine Association, the international conference “Posttraumatic stress: state-of-the-art research and clinical implications for China” was organized in Hangzhou, China, on 17–19 October 2014.

The organizational team was led by Dr Zhonglin Tan, a psychiatrist from The Seventh Hospital of Hangzhou, who has spent a year with Professor Olff's research team in Amsterdam, The Netherlands. With the support of Dr Zhang, Director of The Seventh Hospital of Hangzhou, and with guidance from Professor Olff, Dr Zhonglin Tan successfully created a full program. Over 300 participants, the majority of them from China, enjoyed one full day in both English and Chinese (with simultaneous translation) and the rest of the meeting in Chinese only.

The renowned board members of ISTSS (Kaysen, Stappenbeck, Rhew, & Simpson, 2014; Kim, 2014; Kudler, 2014; O'Donnell, 2014; Olff et al., 2014; Schnyder, 2014) and other international experts (Jongedijk, 2014; Wu, 2014) had volunteered to speak at this first ISTSS conference in China, as part of ISTSS's *global meetings program* (see www.istss.org). Recognizing that trauma is a global issue (Schnyder, 2013), ISTSS is opening doors to parts of the world where, up till now, no ISTSS conferences have been held, being eager to spread knowledge, to collaborate, and to learn from each other, and thus to advance the field of traumatic stress worldwide.

Next to the international speakers, a strong representation of Chinese PTSD experts was seen who gave keynotes on topics ranging from biological stress systems (Bao & Swaab, 2014) web-based interventions (Wang, 2014; Wang & Mearcker, 2014) and post-disaster psychosocial care (Zhang S, 2014) to the exciting field of Chinese traditional medicine for PTSD (Zhang Y-H, 2014).

Apart from keynote lectures, this collection contains the abstracts of a selection of over 100 submissions, the authors of which were awarded for their excellent contribution to the field with a certificate and abstract publication in the *European Journal of Psychotraumatology* (Cao, Wang, Wang, Qing, & Zhang, 2014; Hall, Chen, Wu, Zhou, & Latkin, 2014; Hong, Cao, & Efferth, 2014; Reifels et al., 2014; Teng, Hall, & Li, 2014; Xu et al., 2014).

*Miranda Olff* Editor-in-Chief *Zhonglin Tan* Hangzhou Mental Health Center Hangzhou Seventh Peoples’ Hospital Hangzhou, People's Republic of China
