# Supplementary material for: EJPT abstract book for Posttraumatic stress: state-of-the-art research and clinical implications for China
Source: Eur J Psychotraumatol. 2014 Dec 9;5:10.3402/ejpt.v5.26477. doi: 10.3402/ejpt.v5.26477 (PMC4265135; doi:10.3402/ejpt.v5.26477)
Supplement: Related texts

# Sub article: Social support, oxytocin, and PTSD

**DOI:** 10.3402/ejpt.v5.26513

**Published:** 2014-12-09

**Authors:** Miranda Olff, Saskia B. J. Koch, Laura Nawijn, Jessie L. Frijling, Mirjam Van Zuiden, Dick J. Veltman

**Affiliations:** 1 Department of Psychiatry, Academic Medical Center, University of Amsterdam, Amsterdam, The Netherlands; 2 Arq Psychotrauma Expert Group, Diemen, The Netherlands; 3 Department of Psychiatry, VU University Medical Center, Amsterdam, The Netherlands

**Keywords:** oxytocin, social support, PTSD, trauma, fMRI, neuroimaging, amygdala

## Abstract

**Background:**

A lack of social support and recognition by the environment is one of the most consistent risk factors for posttraumatic stress disorder (PTSD), and PTSD patients will recover faster with proper social support. The oxytocin system has been proposed to underlie beneficial effects of social support as it is implicated in both social bonding behavior and reducing stress responsivity, notably amygdala reactivity (Koch et al., 2014; Olff et al., 2010; Olff, 2012). The amygdala is found to be hypersensitive in people with PTSD.

**Method:**

In order to investigate neurobiological mechanisms underlying potential preventive and therapeutic effects of intranasal oxytocin, we performed a series of fMRI studies (funded with a prestigious NWO TOP grant): BONDS standing for “Boosting Oxytocin after trauma: Neurobiology and the Development of Stress-related psychopathology” in acutely traumatized persons admitted to the emergency department (Frijling et al., 2014); BOOSTER “Boosting oxytocin after trauma: the effects of intranasal oxytocin administration on emotional and motivational processing and neural activity in PTSD” in police officers with and without PTSD.

**Results:**

In this presentation, we present the BOOSTER results on the effects of a single oxytocin administration on amygdala reactivity in response to emotional faces in PTSD patients versus traumatized controls. We found significantly decreased bilateral amygdala reactivity towards emotional faces in PTSD patients compared to traumatized controls.

**Conclusions:**

These promising results call for intervention studies such as studying the effects of medication (oxytocin) enhanced psychotherapy in PTSD patients.

## References

[1] Frijling J. L, Van Zuiden M, Koch S. B, Nawijn L, Goslings J. C, Luitse J. S (2014). Efficacy of oxytocin administration early after psychotrauma in preventing the development of PTSD: Study protocol of a randomized controlled trial. BMC Psychiatry.

[2] Koch S. B, Van Zuiden M, Nawijn L, Frijling J. L, Veltman D. J, Olff M (2014). Intranasal oxytocin as strategy for medication-enhanced psychotherapy of PTSD: Salience processing and fear inhibition processes. Psychoneuroendocrinology.

[3] Olff M (2012). Bonding after trauma: On the role of social support and the oxytocin system in traumatic stress. European Journal of Psychotraumatology.

[4] Olff M, Langeland W, Witteveen A, Denys D (2010). A psychobiological rationale for oxytocin in the treatment of posttraumatic stress disorder. CNS Spectrums.

# Sub article: The concept and treatment of psychological trauma

**DOI:** 10.3402/ejpt.v5.26514

**Published:** 2014-12-09

**Authors:** Harold Kudler

**Affiliations:** Department of Psychiatry and Behavioral Sciences, Duke University, Durham, NC, USA

**Keywords:** psychological trauma, PTSD, theory, treatment, diagnosis, evidence-based interventions, transference, countertransference

## Abstract

Despite a large and rapidly expanding literature on psychological trauma, many fundamental questions remain about its basic nature: Is it a psychological problem or a biological one?; Is it a past event somehow stuck in the present or is it something new which has been triggered and shaped by that event?; Does it reside only within the patient or does it live between the patient and other people (including within the therapeutic relationship)? This presentation will review the history of the concept of psychological trauma and explore the theoretical bases for current evidence-based psychotherapies for PTSD, each of which will be shown to describe psychological trauma as a problem in bringing the past and the present together in memory and cognition. These theories primarily differ on the question of whether a traumatic memory becomes pathogenic, because it cannot be biologically processed or because it must be psychologically avoided. Psychoanalytic concepts of transference and countertransference will be shown to be of practical importance regardless of the type of treatment chosen. If researchers and clinicians can build on what they hold in common rather than become divided by their differences, we can improve our ability to understand and alleviate the effects of psychological trauma.

# Sub article: 311 disaster and mental health countermeasures

**DOI:** 10.3402/ejpt.v5.26515

**Published:** 2014-12-09

**Authors:** Yoshiharu Kim

**Affiliations:** National Center of Neurology and Psychiatry, Tokyo, Japan

**Keywords:** earthquake, tsunami, mental health policy, emergency response, psychosocial support, disaster

## Abstract

On 11 March 2011, a devastating earthquake struck off the coast of Japan, causing blustering tsunami that swept over the northeast coast of the country. Many struggled to evacuate from their homes, schools, and workplaces as 8- to 9-m-tall tsunami rapidly reached the coast within half an hour after the earthquake (Emergency Disaster Response Headquarters). The officials reported a record-breaking magnitude of 9.0 Mw, which made this earthquake the most devastating earthquake in the Japan's history. It had not been long since the previous massive earthquake had hit Kobe in 1995, killing 6,434 people (Japan Meteorological Agency). The author presents the outline of the initial mental-health-care responses at various levels. It has focused on the comprehensive strategies and policies that were intended to cover all the affected areas but has not described the individual countermeasures and reactions in each prefecture and city. The psychological effects of the atomic plant accident in Fukushima has not been mentioned in detail, because the scope of the physiological effect of the accident has not been settled yet and the society is not necessarily ready to deal with the accident as a psychological matter rather than a sociopolitical one. A number of psychiatric professionals are deeply concerned with the psychological and prolonged impact of the accident, including those who are in the Fukushima prefecture and conducting heroic efforts to care for the residents.

# Sub article: Psychosocial recovery after serious injury

**DOI:** 10.3402/ejpt.v5.26516

**Published:** 2014-12-09

**Authors:** Meaghan O'Donnell

**Affiliations:** Department of Psychiatry, University of Melbourne, Melbourne, Australia

**Keywords:** injury, psychosocial recovery, transdiagnostic, early intervention, anxiety, depression, PTSD, agoraphobia, substance use

## Abstract

**Background:**

The 2010 iteration of the Global Burden of Disease statistics (Murray et al., 2012) points to the growing impact of injury and highlights the mounting burden of psychiatric disorder. It is essential to examine the intersection between these two contributors to disease burden.

**Methods:**

The Australian Injury Vulnerability Study collected data of over 1,000 injury patients from their initial hospitalization to 6 years post-injury. Structured clinical interviews were used to diagnose psychiatric disorder and self-report measures for disability and symptom severity.

**Results:**

A wide range of psychiatric disorders developed following injury, which included posttraumatic stress disorder, agoraphobia, depression, and substance use disorders (Bryant, O'Donnell, Creamer, Silove, & McFarlane, 2010). Although prevalence rates for these disorders were generally consistent over time, examination of trajectory data showed that different people had the disorders at different times. Importantly, the data showed that early anxiety, depression, and PTSD symptoms played a significant role in the development of long term disability after injury (Carty, O'Donnell, Evans, Kazantzis, & Creamer, 2011; O'Donnell et al., 2013).

**Conclusions:**

These data support the view that transdiagnostic models for early intervention may be required to address the complex psychiatric disorder trajectories that develop after injury.

## References

[1] Bryant R. A, O'Donnell M. L, Creamer M, Silove D, McFarlane A. C (2010). The psychiatric sequelae of traumatic injury. American Journal of Psychiatry.

[2] Carty J, O'Donnell M, Evans L, Kazantzis N, Creamer M (2011). Predicting posttraumatic stress disorder symptoms and pain intensity following severe injury: The role of catastrophizing. European Journal of Psychotraumatology.

[3] Murray C. J. L, Vos T, Lozano R, Naghavi M, Flaxman A. D, Michaud C (2012). Disability-adjusted life years (DALYs) for 291 diseases and injuries in 21 regions, 1990–2010: A systematic analysis for the Global Burden of Disease Study. The Lancet.

[4] O'Donnell M. L, Varker T, Holmes A. C, Ellen S, Wade D, Creamer M (2013). Disability after injury: The cumulative burden of physical and mental health. Journal of Clinical Psychiatry.

# Sub article: PTSD and loss: preliminary findings from a territory-wide epidemiology study in Hong Kong

**DOI:** 10.3402/ejpt.v5.26517

**Published:** 2014-12-09

**Authors:** Kitty K. Wu

**Affiliations:** Kwai Chung Hospital, Hong Kong, People's Republic of China

**Keywords:** loss, grief, prevalence, trauma, social support, screening

## Abstract

**Background:**

The study examined the prevalence of trauma and posttraumatic stress disorder (PTSD) symptoms among community dwelling Chinese adults in Hong Kong. The relationship of traumatic life events (including loss) and mental health has been investigated.

**Methods:**

The sampling of the collaborative study (HKMMS: Hong Kong Mental Morbidity Survey) adopts a multi-stage stratification approach with the distribution of residential premises in different geographical districts and the relative proportion of private versus public housing units taken into consideration. In Phase I of this study, 4,644 adults were screened for PTSD with the Trauma Screening Questionnaire (TSQ) and Life Event Checklist (LEC), Beck's scales and CIS-R (Revised Clinical Interview Schedule). In Phase II of the study, clinical psychologists conducted the Structured Clinical Interview for DSM Disorders (SCID) for 92 participants (results not reported here).

**Results:**

Among Phase I participants, 65% reported traumatic experience (including 18% who reported personal experience of sudden death of significant others). Age and gender make a difference in traumatic experience. When compared to participants who reported no traumatic experience in the past, participants who reported to have personal experience of sudden death of significant others or other traumatic experiences were found to have higher TSQ scores, higher psychological distress, lower social support (PSS: Multidimensional Scale of Perceived Social Support), and lower life functioning (SOFAS: Social and Occupational Functioning Assessment Scale), *p*<0.001. Findings of hierarchical regression showed that type of trauma (i.e., loss, other trauma, or no trauma) contributed significantly to the prediction of all the mental health indices after demographic and social variables were controlled.

**Conclusions:**

Public education on the association of traumatic experience and psychological health, as well as the monitoring of mental health for at-risk individuals are suggested for early identification of people in need of mental health services.

# Sub article: Proximal relationships between PTSD and drinking behavior

**DOI:** 10.3402/ejpt.v5.26518

**Published:** 2014-12-09

**Authors:** Debra Kaysen, Cynthia Stappenbeck, Issac Rhew, Tracy Simpson

**Affiliations:** Department of Psychiatry & Behavioral Sciences (PBSCI), University of Washington, Seattle, WA, USA

**Keywords:** alcohol abuse, drinking behavior, social support, sexual trauma, victimization, self-efficacy, comorbidity

## Abstract

Co-morbid PTSD and alcohol use disorders are both common and debilitating. However, many of these studies rely on cross-sectional studies that obscure more complex relationships between PTSD and drinking. Event-level studies allow for examination of proximal relationships between PTSD and drinking. Among women (*n*=136 with past sexual victimization, *n*=40 no past trauma history), a two-part mixed hurdle model was used to examine daily PTSD and drinking. On days women experienced more intrusive and behavioral avoidance symptoms, they were more likely to drink. For a 2 SD increase in symptoms, there was a 5% increased likelihood of drinking, and for a 2 SD increase in dysphoric symptoms or negative affect, women drank approximately half drink less. Daily-level coping self-efficacy moderated the association between distress and drinking (IRR=0.91, *p*<0.01). Women who reported less coping drank more as their distress increased on a certain day whereas women who reported more coping drank about the same regardless of distress. Overall, findings suggest that specific PTSD symptoms are associated with higher alcohol use and that these relationships are moderated by daily coping self-efficacy. Implications of these findings for informing models of PTSD/AUD comorbidity, as well as clinical implications will be discussed.

# Sub article: Web-based interventions for traumatized people in mainland China

**DOI:** 10.3402/ejpt.v5.26519

**Published:** 2014-12-09

**Authors:** Jian-Ping Wang, Andreas Maercker

**Affiliations:** 1 School of Psychology, Beijing Normal University, Beijing, People's Republic of China; 2 Department of Psychiatry and Psychotherapy, University of Zurich, Zürich, Switzerland

**Keywords:** Internet interventions, online self-help, e-health, CBT, RCT, efficacy

## Abstract

**Background:**

The Internet is now becoming a new channel for delivering psychological interventions.

**Method:**

This paper reported a first application of web-based intervention in mainland China. It first summarized primary barriers to mental health help-seeking behavior in Chinese society. Then, it introduced the current utilization of the Internet within mental health services in mainland China and discussed how the Internet would help to improve people's help-seeking behaviors. More importantly, it presented main empirical findings from a randomized controlled trial (RCT) which investigated the efficacy of a web-based self-help intervention program (Chinese My Trauma Recovery website, CMTR) for 103 urban and 93 rural traumatized Chinese persons.

**Results:**

The data revealed that 59% urban and 97% rural participants completed the posttest. In the urban sample, data showed a significant group×time interaction in Posttraumatic Diagnostic Scale (PDS) scores (F1,88=7.65, *p*=0.007). CMTR reduced posttraumatic symptoms significantly with high effect size after intervention (F1,45=15.13, Cohen's *d*=0.81, *p*<0.001) and the reduction was sustained over a 3-month follow-up (F1,45=17.29, Cohen's *d*=0.87, *p*<0.001). In the rural sample, the group×time interaction was also significant in PDS scores (F1,91=5.35, *p*=0.02). Posttraumatic symptoms decreased significantly after intervention (F1,48=43.97, Cohen's *d*=1.34, *p*<0.001) and during the follow-up period (F1,48=24.22, Cohen's *d*=0.99, *p*<0.001).

**Conclusions:**

These findings give preliminary support for the short-term efficacy of CMTR in the two Chinese populations. Finally, some implications are given for the future application of web-based interventions for PTSD in mainland China.

# Sub article: Treating intrusions, promoting resilience: an overview of therapies for trauma-related psychological disorders

**DOI:** 10.3402/ejpt.v5.26520

**Published:** 2014-12-09

**Authors:** Ulrich Schnyder

**Affiliations:** Department of Psychiatry and Psychotherapy, Zurich University Hospital, Zurich, Switzerland

**Keywords:** evidence-based interventions, trauma-focused CBT, cognitive therapy, prolonged exposure, CPT, NET, EMDR, BEPP, resilience, mini-interventions, trauma, PTSD

## Abstract

The efficacy of psychotherapeutic approaches in the treatment of posttraumatic stress disorder (PTSD) can be regarded as empirically demonstrated. Overall, effect sizes appear to be higher for psychotherapy than for medication. Many well-controlled trials with a mixed variety of trauma survivors have demonstrated that trauma-focused cognitive-behavioral therapy (TF-CBT) is effective in treating PTSD. Prolonged exposure therapy (PE) is currently seen as the treatment with the strongest evidence for its efficacy. Cognitive therapy (CT) and cognitive processing therapy (CPT), with their stronger emphasis on cognitive techniques, and Eye Movement Desensitization and Reprocessing (EMDR) seem equally effective. More recent developments include brief eclectic psychotherapy for PTSD (BEPP) and narrative exposure therapy (NET). Emerging evidence shows that TF-CBT can successfully be applied in PTSD patients suffering from severe comorbidities such as borderline personality disorder or substance abuse disorder (Schnyder & Cloitre, 2015). There is also a trend towards developing “mini-interventions,” that is, short modules tailored to approach specific problems. Moreover, evidence-based approaches should be complemented by interventions that aim at promoting human resilience to stress. Finally, given the globalization of our societies (Schnyder, 2013), culture-sensitive psychotherapists should try to understand the cultural components of a patient's illness and help-seeking behaviors, as well as their expectations with regard to treatment.

## References

[1] Schnyder U (2013). Trauma is a global issue. European Journal of Psychotraumatology.

[2] Schnyder U, Cloitre M (2015). Evidence-based treatments for trauma-related psychological disorders. A practical guide for clinicians.

# Sub article: The stress systems in depression: a postmortem study

**DOI:** 10.3402/ejpt.v5.26521

**Published:** 2014-12-09

**Authors:** Ai-Min Bao, Dick F. Swaab

**Affiliations:** 1 Department of Neurobiology, Institute of Neuroscience, Zhejiang University School of Medicine, Hangzhou, People's Republic of China; 2 Netherlands Institute for Neuroscience, an Institute of the Royal Netherlands Academy of Arts and Sciences, Amsterdam, The Netherlands

**Keywords:** molecular mechanisms, brain, neurotransmitters, neuromodulators, genetics, HPA axis, sex steroids, oxytocin, orexin

## Abstract

After trauma, depressive disorders are among the most frequent emerging diagnoses. However, although the symptoms of depression are well characterized, the molecular mechanisms underlying this disorder are largely unknown. Factors involved in the heterogeneous pathogenesis of depression include polymorphisms in stress-related genes, gender, age, developmental history, and environmental (traumatic) stressors such as epigenetic factors. These factors may make different parts of the stress-related brain systems more vulnerable to different stressful or traumatic life events or psychological stresses, causing alterations in a network of neurotransmitters and neuromodulators including amines, amino acids, nitric oxide (NO), and neuropeptides, and finally make individuals at risk for depression. The hypothalamo–pituitary–adrenal (HPA) axis has a prominent position in this network. With the postmortem brain material obtained from the Netherlands Brain Bank, we have carried on a series of studies with the aim to elucidate the specific changes in these systems in relation to special subtypes of depression. Our final destination is to set up tailor-made treatment for depressive patients on the basis of his/her developmental history, genetic and epigenetic background, and the vulnerability in particular neurobiological systems. This presentation is a review of our findings of changes in systems of sex steroids, receptors in the hypothalamic paraventricular nucleus, corticotrophin-releasing hormone, orexin, *γ*-aminobutyric acid, and NO in the etiology of depression, in relation to HPA activity, sex differences, and suicide.

# Sub article: Narrative exposure therapy: an evidence-based treatment for multiple and complex trauma

**DOI:** 10.3402/ejpt.v5.26522

**Published:** 2014-12-09

**Authors:** Ruud A. Jongedijk

**Affiliations:** Foundation Centrum '45 and Arq Psychotrauma Expert Group, Diemen, The Netherlands

**Keywords:** NET, narratives, exposure, refugees, complex trauma, PTSD, evidence-based treatment

## Abstract

Narrative exposure therapy (NET) is a recently developed, short-term treatment for patients with a posttraumatic stress disorder (PTSD) as a result of multiple trauma. NET can be applied very successfully in patients with complex trauma complaints (Jongedijk, 2014; Schauer, Neuner, & Elbert, 2011).

An important feature of NET is that trauma processing is never an isolated event but is always embedded in the context of a traumatic event and in the life history as a whole. At the start, the lifeline is laid. The lifeline is made up of a rope, with flowers (happy events), stones (traumatic events), sometimes candles (grief), or recently also sticks for aggressive acts (NET for offenders; see Stenmark, Cuneyt Guzey, Elbert, & Holen, 2014). These symbols are laid down along the rope, in chronological order. Subsequently, in the subsequent therapy sessions the lifeline is processed in chronological order, giving attention to all the important events a person has experienced in his or her life, both the adverse as well as the pleasurable ones. The narration ends with a written testimony.

To date, there is good evidence NET is effective in the treatment of PTSD patients, with support from 18 RCTs (*N*=950). For culturally diverse populations, NET is recommended as the most evidence-based trauma treatment, besides culturally adapted CBT. NET has been investigated in different populations in Africa, Europe, and Asia. In Asia, research has been carried out in Sri Lanka as well as in China. In China, NET was conducted and investigated with survivors of the Sichuan earthquake (Zang, Hunt, & Cox, 2013, 2014). NET is understandable, even appealing and also supportive for patients with multiple trauma. In this presentation, the treatment principles and the practice of NET will be explained.

## References

[1] Jongedijk R. A (2014). Levensverhalen en psychotrauma. Narratieve exposure therapie in theorie en praktijk.

[2] Schauer M, Neuner F, Elbert T (2011). Narrative Exposure Therapy: A short-term intervention for traumatic stress disorders.

[3] Stenmark H, Cuneyt Guzey I, Elbert T, Holen A (2014). Gender and offender status predicting treatment success in refugees and asylum seekers with PTSD. European Journal of Psychotraumatology.

[4] Zang Y, Hunt N, Cox T (2013). A randomized controlled pilot study: The effectiveness of narrative exposure therapy with adult survivors of the Sichuan earthquake. BMC Psychiatry.

[5] Zang Y, Hunt N, Cox T (2014). Adapting narrative exposure therapy for Chinese earthquake survivors: A pilot randomised controlled feasibility study. BMC Psychiatry.

# Sub article: The treatment and research for posttraumatic stress disorder with Chinese medicine

**DOI:** 10.3402/ejpt.v5.26524

**Published:** 2014-12-09

**Authors:** Yong-Hua Zhang

**Affiliations:** The Seventh Hospital of Hangzhou, Hangzhou, People's Republic of China

**Keywords:** TCM, PTSD, trauma, depression, research

## Abstract

There is no disease called posttraumatic stress disorder (PTSD) in traditional Chinese medicine (TCM). However, Huangdi's Canon of Medicine written in about 200 BC, one of the most famous TCM classics, recorded diseases with similar etiology, pathogenesis, and clinical symptoms. Moreover, contemporary TCM also attaches great importance to diseases caused by trauma. Especially after 2008, there is a mini-rush of study on PTSD as a result of Sichuan earthquake. Referring to ancient and modern literature, we summarize the TCM treatment of PTSD and wish to contribute to the further study on TCM remedy for PTSD.

# Sub article: Localization of post-disaster psychosocial care in China

**DOI:** 10.3402/ejpt.v5.26525

**Published:** 2014-12-09

**Authors:** Sujuan Zhang

**Affiliations:** National Disaster Reduction Center of Ministry of Civil Affairs of China, Beijing, People's Republic of China

**Keywords:** disaster relief, psychosocial care, spiritual pain, culturally sensitive, mental health policy, research

## Abstract

Disaster is not independent of society and culture and always happens in specific cultural and social contexts. Cultural and social characteristics influence the responses of people affected by disaster, as well as the process of disaster relief.

As one of the countries in the world that suffer most from natural disasters, various ethnic groups in China vary greatly in psychology and behavior characteristics after major disasters due to different geographical environments and economic and political conditions. To launch an effective post-disaster psychosocial care, 1) it is necessary to consider how to satisfy material, health, and other fundamental biological needs of affected people; 2) it is necessary to relieve disaster victims of their mental pain (spiritual in Chinese) and help them restore their psychological health; 3) it is necessary to revitalize the seriously unbalanced communities affected by disasters so that these communities would burst with vitality again. In addition, it is necessary to take specific ethnic and regional culture into account when helping people in these areas gradually achieve social adaptation and cultural identification. All these require us to intensify our efforts in the following four aspects: 1) to strengthen legislation and institutional construction in this field; 2) to help citizens master the most fundamental psychological principles and methods of coping with disasters to enable timely self-aid and mutual-aid; 3) to build a national database of the post-disaster psychosocial care teams; 4) to continue the research on disaster psychology, so as to provide a scientific basis as well as techniques and methods for implementing disaster relief efforts in a scientific way.

# Sub article: Adherence to a web-based intervention program for traumatized persons in mainland China

**DOI:** 10.3402/ejpt.v5.26526

**Published:** 2014-12-09

**Authors:** Zhiyun Wang

**Affiliations:** Department of Psychology, Wuhan University, Wuhan, People's Republic of China

**Keywords:** e-Health, online interventions, web-based, Chinese My Trauma Recovery, adherence, depression, PTSD, psychotrauma, self-efficacy

## Abstract

**Background:**

This paper investigated adherence to a self-help web-based intervention for PTSD (Chinese My Trauma Recovery, CMTR) in mainland China and evaluated the association between adherence measures and potential predictors, for example, traumatic symptoms and self-efficacy.

**Methods:**

Data from 56 urban and 90 rural trauma survivors were reported who used at least one of the seven recovery modules of CMTR.

**Results:**

The results showed that 80% urban users visited CMTR four or less days and 87% rural users visited CMTR for 5 or 6 days. On average, urban users visited 2.54 (SD=1.99) modules on the first visiting day and less from the second day; rural users visited 1.10 (SD=0.54) modules on the first visiting day, and it became stable in the following days. In both samples, depression scores at pre-test were significantly or trend significantly associated with the number of visited web pages in the relaxation and professional help modules (*r*=0.20–0.26, all *p*<0.14); traumatic symptom scores at pre-test significantly or trend significantly correlated to the number of visited web pages in the relaxation, professional help, and mastery tools modules (*r*=0.20–0.26, all *p*<0.10). Moreover, urban users’ coping self-efficacy scores at pre-test significantly or trend significantly related to the number of visited web pages in the relaxation, professional help, social support, and mastery tool modules (*r*=0.20–0.33, all *p*<0.16).

**Conclusions:**

These findings suggest that individuals tend to focus on one or two recovery modules when they visit CMTR, and the number of web pages visited during the intervention period relates to users’ traumatic and depressive symptoms and self-efficacy before intervention.

# Sub article: Patterns and predictors of primary mental health service use following bushfire and flood disasters

**DOI:** 10.3402/ejpt.v5.26527

**Published:** 2014-12-09

**Authors:** Lennart Reifels, Bridget Bassilios, Matthew Spittal, Kylie King, Justine Fletcher, Jane Pirkis

**Affiliations:** Centre for Mental Health, Melbourne School of Population and Global Health, The University of Melbourne, Melbourne, Australia

**Keywords:** primary mental healthcare, access to care, service use, bushfire, flood, disasters

## Abstract

**Background:**

Mental health care services play an important role following disasters (Reifels et al., 2013). The aim of this study is to examine patterns and predictors of primary mental health care service use, following two major Australian natural disaster events.

**Method:**

Utilizing referral and session data from a national minimum dataset, descriptive and regression analyses were conducted to identify levels and predictors of the use of the Access to Allied Psychological Services (ATAPS) program over a 2-year period following two major Australian bushfire and flood/cyclone disasters. Predictor variables examined in negative binomial regression analysis included consumer (age, gender, household structure, previous mental health care history, and diagnosis) and event characteristics (disaster type).

**Results:**

The bushfire disaster resulted in significantly greater service volume, with more than twice the number of referrals and nearly three times the number of sessions. Service delivery for both disasters peaked in the third quarter. Consumers affected by bushfires, diagnosed with depression, anxiety, or both of these disorders utilized sessions at significantly higher rates.

**Conclusions:**

The substantial demand for primary mental health services following disaster can vary with disaster type. Disaster type and need-based variables as key drivers of service use intensity indicate an equitable level of service use. Established usage patterns assist with estimating future service capacity requirements. Flexible referral pathways can enhance access to disaster mental health care. Future research should examine the impact of program- and agency-level factors on mental health service use and factors underpinning treatment non-adherence following disaster.

## References

[1] Reifels L, Pietrantoni L, Prati G, Kim Y, Kilpatrick D, Dyb G (2013). Lessons learned about psychosocial responses to disaster and mass trauma: An international perspective. European Journal of Psychotraumatology.

# Sub article: The association between social resources and depression among female migrants affected by domestic violence

**DOI:** 10.3402/ejpt.v5.26528

**Published:** 2014-12-09

**Authors:** Pan Teng, Brian J. Hall, Ling Li

**Affiliations:** 1 Faculty of Medical Statistics and Epidemiology, School of Public Health, Sun Yat-sen University, Guangzhou, People's Republic of China; 2 School of Public Health, Sun Yat-sen Center for Migrant Health Policy, Guangzhou, People's Republic of China; 3 Department of Psychology, University of Macau, Taipa, Macau (SAR), People's Republic of China

**Keywords:** Intimate partner violence, depression, social resources, migration, China

## Abstract

**Background:**

Interpersonal violence (IPV) is associated with higher risk of depression. Female Chinese rural-to-urban migrants may experience greater depression following exposure to IPV due to lack of social support and integration within their receiving communities. The current study estimated the prevalence of IPV among rural-to-urban migrants in Guangzhou, China, and evaluated the moderating effects of social resources on migrant's depression symptoms.

**Method:**

We recruited 1,368 women (1,003 migrants and 365 local-born) of childbearing age from population and family planning centers in two districts using a quota sampling method matched to the 2012 population census. Chinese versions of the Conflict Tactics Scale 2 Short Form, Center for Epidemiological Studies Depression Scale and the Social Support Rating Scale measured IPV, depression, and social support. Social integration was measured with a locally derived scale.

**Results:**

Migrants reported a similar prevalence for IPV (41.20%) to local women (39.20%). Bivariate comparisons demonstrated that migrants reported greater depression (11.8±8.9 vs. 10.0±8.8, *t*=−3.27, *p*<0.001) and less social support (22.2±5.1 vs. 27.1±5.5, *t*=14.84, *p*<0.001). Regression analysis indicated that the effect of violence on depression symptoms for migrant women was moderated by social integration. Women who experienced violence and had greater integration in their community reported less depression than women who experienced violence but reported less social integration.

**Conclusion:**

A high prevalence of IPV was reported in our sample. Social integration is a key risk factor for migrant mental health. Social services aimed to reduce IPV and integrate migrants in their new communities are needed.

# Sub article: Prevalence of potentially traumatic events, depression, alcohol use, and social network supports among Chinese migrants: an epidemiological study in Guangzhou, China

**DOI:** 10.3402/ejpt.v5.26529

**Published:** 2014-12-09

**Authors:** Brian J. Hall, Wen Chen, Yan Wu, Fangjing Zhou, Carl Latkin

**Affiliations:** 1 Department of Psychology, University of Macau, Taipa, Macau (SAR), People's Republic of China; 2 Faculty of Medical Statistics and Epidemiology, School of Public Health, Sun Yat-sen University, Guangzhou, People's Republic of China; 3 School of Public Health, Sun Yat-sen Center for Migrant Health Policy, Guangzhou, People's Republic of China; 4 Department of Health Behavior and Society, Johns Hopkins Bloomberg School of Public Health, Baltimore, USA; 5 Department of Epidemiology, Johns Hopkins Bloomberg School of Public Health, Baltimore, MD, USA

**Keywords:** depression, alcohol abuse, social resources, migration, China

## Abstract

**Background:**

Addressing the health needs of Chinese migrants is a critical public health concern. Epidemiological studies are needed to establish the prevalence of potentially traumatic events (PTEs) and common mental disorders among Chinese migrants and identify protective community and social resources.

**Method:**

Utilizing random household sampling, we are in the process of recruiting a representative sample of Chinese adults (*N*=1,000) in two districts home to a large number of internal migrants. Data are collected using face-to-face interviews and participant self-report methods. Chinese versions of the Life Events Checklist, Alcohol Use Disorders Identification Test, Patient Health Questionnaire and the Social Support Rating Scale measured exposure to PTEs, alcohol use disorder, depression, and social support networks.

**Results:**

Preliminary results indicate a high proportion (68%) of the sample was exposed directly or indirectly to at least one PTE. The most commonly reported events were transportation accidents (43%), natural disasters (39%), and physical assault (26%). A total of 17% of the sample reported drinking consistent with having an alcohol use disorder. Moderate or severe depression was reported by 9% of the sample. The majority (75%) reported having three or more people to rely on for support, and 41% reported active participation in civic groups. Despite these strengths, only half the sample reported having trust in their community.

**Conclusion:**

Preliminary evidence from this population-level survey indicates high exposure to PTEs and a high potential burden of alcohol use disorders. The role of social networks will be explored as potentially useful for community-based intervention development.

# Sub article: Oxytocin is associated with PTSD's anxious arousal symptoms in Chinese male earthquake survivors

**DOI:** 10.3402/ejpt.v5.26530

**Published:** 2014-12-09

**Authors:** Chengqi Cao, Li Wang, Richu Wang, Yulan Qing, Jianxin Zhang

**Affiliations:** 1 Key Laboratory of Mental Health, Institute of Psychology, Chinese Academy of Sciences, Beijing, People's Republic of China; 2 Institute of Psychology, University of Chinese Academy of Sciences, Beijing, People's Republic of China

**Keywords:** PTSD, oxytocin, phenotypic model, association study

## Abstract

**Background:**

Posttraumatic stress disorder (PTSD) is a complex and severe mental disorder triggered by exposure to an extraordinarily traumatic event. Human and animal studies have implied the functional role of the oxytocin system in the development of PTSD (Cochran, Fallon, Hill, & Frazier, 2013; Koch et al., 2014; Olff, 2012). Specification of the role of the oxytocin system in the emergence and progression of PTSD symptomatology would provide evidence to inform both theory and clinical practice.

**Methods:**

This study examined the association between oxytocin serum levels and PTSD symptoms. A total of 106 Chinese male adults who suffered from the deadly 2008 Wenchuan earthquake participated in this study. PTSD symptoms were measured with PTSD Checklist for DSM-5 (PCL-5), and serum oxytocin level was determined with ELISA oxytocin kits.

**Results:**

The mean score on the PCL-5 was 19.30 (SD=14.50, range: 1–65) in this sample. The mean oxytocin level was 101.59 pg/ml (SD=55.89, range: 31.50–286.71). The results indicated that although the oxytocin was not associated with total PTSD symptoms, it was associated with PTSD's anxious arousal symptoms.

**Conclusion:**

These findings support that the oxytocin may play an important functional role in the development of PTSD and contribute to the extant knowledge on the genetic basis of the PTSD symptoms.

## References

[1] Cochran D. M, Fallon D, Hill M, Frazier J. A (2013). The role of oxytocin in psychiatric disorders: A review of biological and therapeutic research findings. Harvard Review of Psychiatry.

[2] Koch S. B, Van Zuiden M, Nawijn L, Frijling J. L, Veltman D. J, Olff M (2014). Intranasal oxytocin as strategy for medication-enhanced psychotherapy of PTSD: Salience processing and fear inhibition processes. Psychoneuroendocrinology.

[3] Olff M (2012). Bonding after trauma: On the role of social support and the oxytocin system in traumatic stress. European Journal of Psychotraumatology.

# Sub article: Posttraumatic stress disorder among earthquake survivors of the Wenchuan area (Sichuan, China)

**DOI:** 10.3402/ejpt.v5.26531

**Published:** 2014-12-09

**Authors:** Chunlan Hong, Jingming Cao, Thomas Efferth

**Affiliations:** Department of Pharmaceutical Biology, Institute of Pharmacy and Biochemistry, Johannes Gutenberg University, Mainz, Germany

**Keywords:** PTSD, earthquake, biomarker, phytotherapy, intervention

## Abstract

**Background:**

On May 12, 2008, an earthquake with a power of 8.0 M on the Richter scale occurred in the Wenchuan County of Sichuan Province in southwest China, which was unprecedented in magnitude and aftermath. Approximately 70,000 people were killed and nearly 20,000 went missing. The earthquake caused a wide number of mental and physical health outcomes among survivors, and posttraumatic stress disorder (PTSD) was one of the most commonly studied.

**Methods:**

We conducted a systematic overview to assess research achievements about PTSD in the past 6 years after the Wenchuan earthquake, including symptoms and risk factors about PTSD among Wenchuan earthquake survivors, as well as research developments in genetics, molecular biology, and treatment of PTSD.

**Results:**

The large body of research conducted after the Wenchuan earthquake suggests that the burden of PTSD among persons with high exposure was substantial. Adolescents and adults were among the most studied populations with high prevalence rates. Phytotherapy with Chinese herbs as well as acupuncture were rarely studied as of yet, although published data indicated promising therapy effects. Genome-wide microarray technologies are widely used in experimental mice and rat models to study PTSD mechanisms as well as in patients suffering from PTSD and other psychosomatic disorders to search for novel biomarkers and to monitor the effectiveness of treatment interventions.

**Conclusion:**

Using genomic and transcriptomic technologies, our future research will focus on the efficacy and safety of Chinese medicine to find potential interventions and effective treatments of PTSD.

# Sub article: Stressful parental bonding exaggerate the functional and emotional disturbances of primary dysmenorrhea

**DOI:** 10.3402/ejpt.v5.26532

**Published:** 2014-12-09

**Authors:** Kai Xu, Liuxi Chen, Lingyun Fu, Hongjing Mao, Jian Liu, Wei Wang

**Affiliations:** Department of Clinical Psychology and Psychiatry, School of Public Health, Zhejiang University College of Medicine, Hangzhou, People's Republic of China

**Keywords:** parental bonding, primary dysmenorrhea, trauma, attachment, family relationships, painful period, emotional disturbance

## Abstract

**Background:**

Some evidence suggests that women with primary dysmenorrhea (or painful period) often have traumatic experience with parental attachments, but the exact relationship between styles of the parental bonding and the detailed aspects of the disorder is unclear.

**Methods:**

From university-student women, we invited 50 primary dysmenorrhea patients and 111 healthy volunteers to undergo tests of the functional and emotional measure of dysmenorrhea (FEMD), the Family Relationship Questionnaire (FRQ), and the visual analog scale for the pain intensity experienced.

**Results:**

Besides the high scores of the FEMD functional and emotional scales, the dysmenorrhea patients also scored significantly higher than the healthy controls on the FRQ scales of paternal dominance and maternal abuse. In patients, the FEMD Emotional scale was negatively predicted by the Paternal Freedom Release scale and the FEMD functional scale was positively predicted by the Maternal Dominance scale.

**Conclusion:**

Inappropriate parental bonding or chronic traumatic attachment styles have respective relationships with the functional and emotional disturbances experienced by the primary dysmenorrhea patients.
